# SRT-Joy – computer-assisted self-regulation training for obese children and adolescents: study protocol for a randomized controlled trial

**DOI:** 10.1186/s13063-015-1078-2

**Published:** 2015-12-10

**Authors:** Petra Warschburger

**Affiliations:** Department Psychology, Counselling Psychology, University of Potsdam, Karl Liebknecht-Str. 24-25, 14476 Potsdam, Germany

**Keywords:** Obesity, Randomized-controlled trial, Computer-assisted self-regulation training, Children, Adolescents, Weight

## Abstract

**Background:**

Obesity is not only a highly prevalent disease but also poses a considerable burden on children and their families. Evidence is increasing that a lack of self-regulation skills may play a role in the etiology and maintenance of obesity. Our goal with this currently ongoing trial is to examine whether training that focuses on the enhancement of self-regulation skills may increase the sustainability of a complex lifestyle intervention.

**Methods/Design:**

In a multicenter, prospective, parallel group, randomized controlled superiority trial, 226 obese children and adolescents aged 8 to 16 years will be allocated either to a newly developed computer-training program to improve their self-regulation abilities or to a placebo control group. Randomization occurs centrally and blockwise at a 1:1 allocation ratio for each center. This study is performed in pediatric inpatient rehabilitation facilities specialized in the treatment of obesity. Observer-blind assessments of outcome variables take place at four times: at the beginning of the rehabilitation (pre), at the end of the training in the rehabilitation (post), and 6 and 12 months post-rehabilitation intervention. The primary outcome is the course of BMI-SDS over 1 year after the end of the inpatient rehabilitation. Secondary endpoints are the self-regulation skills. In addition, health-related quality of life, and snack intake will be analyzed.

**Discussion:**

The computer-based training programs might be a feasible and attractive tool to increase the sustainability of the weight loss reached during inpatient rehabilitation.

**Trial registration:**

The present study protocol was registered on 13 July 2015 at German Clinical Trials Register: DRKS00007879.

## Background

Obesity remains the most serious health threat worldwide. To take the example of Germany, approximately 6.1 % of the children and adolescents are obese [1]. Childhood obesity is already associated with various adverse outcomes such as hypertension, dyslipidemia, hyperinsulinemia and prediabetic state. In addition, obese children and adolescents exhibit reduced psychosocial functioning, especially those seeking treatment [2, 3]. Obesity is not only highly prevalent in childhood, but substantial forward tracking of obesity into adulthood also exists [2]. The economic burden of obesity and its related comorbidities is enormous. Thus, obesity is associated with 0.7 to 2.8 % of health-related expenditure linked to society; obese individuals cause 30 % more direct costs compared to normal-weight individuals [4]. In addition, an increased rate of sick days [5] and reduced opportunities for higher education and on the labor market [6–8] are reported.

Today’s living conditions and food habits in children and adolescents are generally characterized by a combination of increased energy consumption and sedentary lifestyles, which strongly favor the emergence of overweight and obesity. In this obesogenic environment, children and adolescents are particularly challenged to develop and maintain a high “resistance” against the ready availability of food.

Therefore, self-regulatory skills are considered to be a relevant factor in the formation and maintenance of obesity. Self-regulation refers to automatic and conscious processes that enable individuals to control their thoughts, impulses, emotions, and attention, as well as their performances [9]. It includes, among other things, the ability to postpone rewards, to pursue long-term goals or suppress impulses (so-called inhibitory control). Obese children exhibit deficits in various aspects of self-regulation compared to normal-weight children (see also reviews by [10–13]). It is challenging for overweight children to stop an activity that is already mentally planned (for example, [14, 15]) or to postpone rewards, especially when it comes to food-related stimuli (for example, [16, 17]). A low amount of self-regulatory skills has been proven as a predictor of weight increase in prospective studies. Pauli-Pott, Albayrak, Hebebrand, and Pott [18] could show that a lack of inhibitory control will negatively impact a healthy weight development especially in younger children (age 8). In addition, Tsukayama, Toomey, Faith, and Duckworth [19] suggest in their longitudinal study that children who have a high impulse control and enhanced ability to delay gratification at the age of 9 years are significantly less likely to be overweight at the age of 15 compared to their less controlled peers. They conclude that the ability to self-regulate and control enables individuals to maintain a healthy weight status. Similar findings have been reported by Seeyave et al. [20].

The ability to maintain the weight at a healthy weight range in the long term is the ultimate goal of intervention programs. Overall, the empirical data on the effectiveness of treatment programs in childhood and adolescence is cautiously optimistic: meta-analyses [21–24] have suggested that, in most cases, a small but clinically meaningful weight loss can be achieved. However, many studies only included short-term follow-up periods, and the long-term maintenance of weight changes presents the major challenge. Even in the area of medical inpatient rehabilitation, an important healthcare forum for the treatment of obese children and adolescents in Germany (30 % of obese children are treated in these settings [25]), recent data indicate that approximately 20 % of children and adolescents had already exceeded their relative standardized BMI-score (BMI-SDS) 1 year after the end of rehabilitation compared to its beginning (Warschburger P, Kröller K, Haerting J, Unverzagt S, Egmond-Frohlich A van: Empowering parents of obese children (EPOC): A randomized-controlled trial on additional long-term weight effects of a parent training, submitted). Strategies to ensure weight-loss maintenance are therefore urgently needed. A computer-based self-regulation training could represent a promising approach. In the field of research on alcoholism, positive effects of such an additional training program have been reported. Alcoholic and nonalcoholic pictures are presented on the computer screen and the task is to either to pull or push the joystick in order to enlarge or decrease the size of the picture. Wiers, Eberl, Rinck, Becker, and Lindenmeyer [26] trained the rejection (avoidance) of alcoholic beverages in combination with an approach response of nonalcoholic beverages during four computer sessions of 15 min each. The authors could show that the rejection of alcoholic beverages can be learned and that positive implicit attitudes toward alcohol decreased after the training. Data from the 1-year follow-up underscore the clinical relevance of the training: a significantly lower relapse rate in the training compared to the control group was observed.

Taken together, research findings suggest a lower expression of self-control skills and greater attention on food-related stimuli for overweight children. Evidence suggests that self-control and performance skills can be used successfully for training during childhood and adolescence [27]. Henceforth, based on the promising results of an approach-avoidance training for adults, self-regulation training will be adapted for overweight children and adolescents in order to train their avoidance skills when confronted with high-caloric food stimuli. Evaluation of short- and long-term effects of the training on behavioral tendencies and weight development will be studied in a multicenter, placebo-controlled study. Given the common cognitive and neural mechanisms of alcohol and “overeating” [28], it is assumed that a nutrition-specific version of the “Approach-Avoidance Training” (AAT) can also be successfully applied in the context of obesity treatment.

## Methods/Design

### Study design

**Fig. 1:**
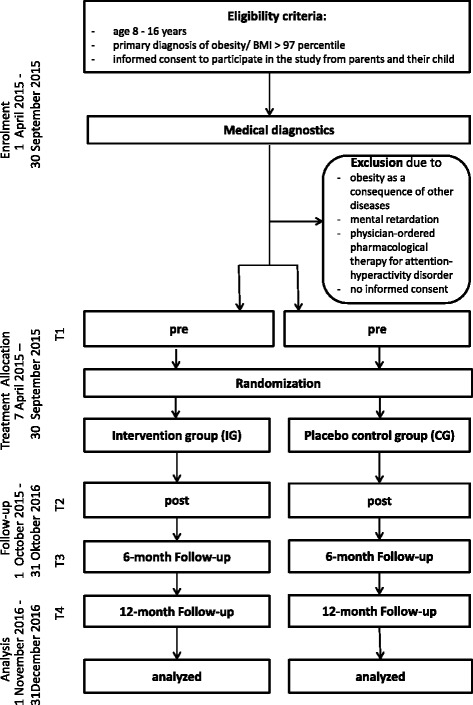
Trial flow chart

The Self-Regulation Training Joystick (SRT-Joy) is a multicenter, prospective, parallel group, randomized-controlled superiority trial for the evaluation of the efficacy of a computer-assisted individual self-regulation training compared to a placebo control group. Depending on their intervention allocation, children play either the SRT-Joy computer task or the placebo training. In order to obtain information on the transfer of increased self-regulation skills to everyday life, two follow-up measurements after the end of the inpatient rehabilitation stay will be realized. Hence, the four time points for measurement are given below: start of the child’s inpatient stay and beginning of the computer training (T1), end of the child’s computer training during the inpatient stay (T2), 6 months after the child’s inpatient stay (T3), and 12 months after the child’s inpatient stay (T4). The trial flow is depicted in Fig. 1.

### Study setting

This study is being conducted in four study centers, all of which are inpatient medical rehabilitation facilities (Bad Kreuznach, Bad Orb, Idar Oberstein, and Sylt). These clinics were chosen because of their specialization in the treatment of childhood obesity and delivering their intervention according to the national guidelines. Participating study centers and investigators are fully listed in the German Clinical Trials Register (DRKS00007879).

### Recruitment

After initiation of the study centers, the 12-month recruitment phase of this ongoing trial started in March 2015. Eligible subjects will be recruited consecutively at their arrival at the clinic. A study coworker will inform the parents and their children about the contents and aims of the study and ask for written informed consent. Participation is voluntary and parents and children can withdraw informed consent at any time without any disadvantages for their treatment within the inpatient treatment facility.

### Participants

In order to ensure a high representativeness of our study results, only a few inclusion and exclusion criteria were defined as listed below:

#### Inclusion criteria

Inclusion criteria are as follows:

1. aged 8 to 16 years at recruitment,
2. obesity as defined by body mass index (BMI) exceeding the 97th percentile [29] based on a medical examination on arrival in the clinic, and
3. informed consent to participate in the study from both the parents and their child.

#### Exclusion criteria

Exclusion criteria include the following:

1. secondary obesity as a consequence of other diseases (such as endocrine or genetic diseases; determined by medical examination),
2. mental retardation,
3. physician-ordered pharmacological therapy for attention-deficit-hyperactivity disorder, or
4. obligatory treatment plans that interfere with the participation in the computer training.

Prior to study inclusion, children will undergo a thorough medical examination.

### Sample size and enrolment

So far, there are no studies that have conducted an AAT on nutrition in children. Therefore, a referral upon existing data could not be used in the power calculation. In total, we only expect small differences between the groups for the primary outcome (age- and gender-specific BMI-SDS course after 6 to 12 months following the intervention), because the AAT will only be an additional offer for children and adolescents within the multidisciplinary rehabilitation. Comparable effect sizes are also assumed for food intake and weight-related quality of life. On the other hand, we expect average effect sizes for the employed self-control measures as secondary outcomes because the AAT is a specific training of these skills. The power calculation was performed with the program *g power 3.1* [30] in several steps to take into account these considerations. The power was set to .8 and the alpha error to .05. For a small effect (f = .2), 199 subjects are needed, with 99/ 100 per group for an analysis of covariance (ANCOVA) that includes three covariates (age, gender, and educational background). Because the data will be primarily analyzed on the basis of an intention-to-treat approach, only a small number out of the 13 patients per group were taken into consideration (see [22]). A subsequent post-hoc compromise analysis (considering differing effect sizes for primary and secondary outcomes) has confirmed that this sample size (n = 226) is sufficient to depict small effects with a power of .9. On that basis the following will occur:

1. n > 600 will be assessed for eligibility,
2. n = 226 will be allocated to trial, and
3. an intention-to-treat approach (ITT) will be conducted on n = 226 (the complete analysis should include n = 199 participants).

To prevent selective drop out and incomplete outcome data, all participants will be reimbursed for completely filling in the questionnaires in the follow-up period and will receive cheerful mail on special occasions (birthday, Easter or Christmas greetings). In addition, the family doctors will receive a monetary incentive for providing anthropometric data (weight and height).

### Intervention and controls

#### Basic interventions for all children

The inpatient stay usually encompasses a period of 4 weeks. During their stay, all children and adolescents will participate in a multidisciplinary lifestyle intervention, which includes cognitive-behavioral group training, nutrition education, and diet modification, as well as activity sessions several times a week. This complex treatment approach focuses on the increase in physical activity, the decrease in sedentary behavior, the change in eating habits, and the relative reduction of weight. The AAT treatment will be implemented as a supplemental treatment offer of the clinics.

#### SRT-Joy Approach-Avoidance-Training: intervention

Children and adolescents far too often eat high-caloric snacks and too seldom vegetables [31]. Therefore, they should learn, in the context of the computer-based training, to push high calorie snacks away and to pull vegetables with the joystick toward themselves. In a pilot study, these foods have been tested on their familiarity and have been matched according to their color. All foods are presented on white either square or round plates. Children are instructed to use the approach response with the round plate (pull) and the avoidance response (push) with the square one using the joystick. Thereby, the vegetables are always located on the round plates and the snacks on the square plates (implicit approach-avoidance training). Contingent with their actions, the photos on the screen get either larger or smaller. A short instruction phase ensures that the children have understood the instructions. This phase is followed by a test trial (pre-test data) and the training phase (including six training blocks). In each training session, the children will view 120 pictures. Compatible (vegetables on round plates, snacks on square plates) and incompatible stimuli are balanced per trial. The training phase is followed by an assessment of the post-data and a booster training. At the end of each training session, the children receive a feedback on their error rate and their reaction time. The training sessions take approximately 15 to 30 min each and are conducted during 3 consecutive days followed by a pause of 4 days over the course of the first 2 weeks of rehabilitation (six appointments).

#### Placebo: control

The children and adolescents of the control group participate in an identical number of training sessions with the same structure as the intervention group. In contrast with the intervention group, children will approach and avoid both (high-calorie snacks and vegetable) at the same rate so that no learning effect occurs.

#### Measures of intervention attendance, treatment integrity and standardization

The intensity of treatment and compliance can be assessed by the PC, as the times and dates of practice are logged, as soon as the children log in with their code (the training times of child are used as a marker of compliance). In addition, the clinics document the other treatment approaches that were carried out. The standardization of the intervention and treatment integrity is given inherently by training on the PC.

### Hypotheses

In the first step, we will examine the trainability of the avoidance of snacks. Thereby, we assume that the children in the intervention group show lower contingent reaction times (pull the vegetable and push the snacks away) in comparison to the control group (see [26]). Since we postulate that the improvement of the children’s ability to self-regulate is an essential determinant for their sustained weight loss, the efficacy of our newly developed computer training program has to be tested in the first step. Evidence of the training effect is the basis for the testing of the further hypotheses.

The primary hypothesis postulates that children in the intervention group will experience a more sustained reduction of their BMI-SDS at the 6- and 12-month follow-up than children in the control group. Secondary hypotheses postulate that children in the intervention group - compared to the placebo control group - will experience a more pronounced and sustained improvement in their self-regulation skill. We further expect a more pronounced and sustained increase in the quality of life among the intervention group as well as a healthier eating behavior (in terms of lower snack consumption). In an exploratory manner, we will explore whether children with a more pronounced binge eating behavior (indicating more self-regulation problems) will profit more from the approach-avoidance training. In contrast, however, we expect children with more pronounced psychosocial strain (internalizing and/or externalizing behaviour problems) will profit less from our intervention than children exhibiting no psychosocial problems.

### Measures

In the SRT-Joy, the AAT registers the reaction times of the children as they use approach-pull movements and avoid-push movements toward the images shown on the screen. In a pilot study, we could already provide evidence that children showed improved reaction times after six training sessions. In line with the other studies [26], we will calculate different indicators: reaction times for snacks (compatible and incompatible reactions), reaction times for vegetables and a so-called compatibility index (reaction times (compatible tasks) – reaction times (incompatible tasks) [26]). The compliance with the treatment protocol can be directly assessed via log-in times of the children. The acceptance of the computerized treatment approach will be assessed via a self-constructed questionnaire at the end of the training. At the beginning and end of each session, the degree of hunger, appetite, and the current mood will be assessed as possible confounding factors.

The Implicit Association Test (IAT) is used to capture the attitude toward snacks. The task is to assign a positive or a negative category to the images and words presented on the screen. The IAT [32] is based on the assumption that when there is a positive association between the positive category (here: “good”) and the target object (here: snacks), the reaction time is shorter (in this example: category “good” and snack image), whereas in a pairing of the negative category (“bad”) and the target object (snacks), the reaction is assumed to be slower because an implicit positive “bias” exists toward snacks.

As a behavioral indicator for the child’s inhibition capacity, a Stroop test is used. Color words are presented on the screen either in congruent (the word “red” typed in red-colored letters) or incongruent (the word “red” typed in blue) writing colors. Children under the age of 12 will be asked to fill in a paper-pencil Fruit Stroop test (images of fruits in different colors; [33]), which works on the same principles as the Stroop test conducted on the computer. The color either corresponds to the actual color of the fruits or not (example congruent: yellow banana, incongruent example: blue banana). There is evidence that the Stroop Test paradigm is a reliable and valid paradigm for assessing the inhibitory control of children (for example, [34]).

The dot probe task is a measure of selective attention. During this computer task, two stimuli (for example, words or images) are simultaneously presented on the screen. One stimulus corresponds to a particular target concept (food), and the other stimulus is neutral. Subsequently, one of the two stimuli is replaced by a dot. The participant is instructed to respond as quickly as possible to the position of the dot by a specific keystroke. Children with a selective attention to food stimuli are expected to react more quickly when the dot replaces the food stimulus compared to the neutral stimulus (for example, [35]).

These computerized mainly food-related behavioural tests will be conducted at the beginning (T1) and end (T2) of the inpatient stay. On the basis of these data, whether the SRT-Joy Training leads to an immediate improvement of the self-regulation abilities of the overweight children (training effect) will be analyzed.

### Primary and secondary outcomes

The primary outcome of the study is the BMI-SDS course, that is, the sustained weight loss after the intensive inpatient lifestyle intervention. Weight and height data of the children will be measured at the beginning and the end of their rehabilitation stay by means of a standard beam scale (accurate to 100 g) and a calibrated stadiometer (accurate to 1 cm). In order to account for sex and age differences, a BMI-SDS will be calculated [29]. In order to receive unbiased anthropometric data at the follow-ups as well, the children will be asked to visit their family physician, who will be blind to trial-group assignment and the study goals. The families (and their physicians) will be reminded several times (by mail and telephone) and reimbursed for their efforts in order to decrease attrition bias.

The secondary outcome is the child’s ability to self-regulate. Self-regulation encompasses conscious as well automatic efforts to achieve long-term goals [9]. In order to assess conscious aspects of self-regulation skills, the Brief Self-Control Scale (BSCS) will be administered. The BSCS by Tangney, Baumeister, and Boone [36] was translated into German and adapted for children, using a multistage process. The 13 items (for example, “I am good at resisting temptations”) will be rated on a five-point Likert scale from “strongly disagree” to “agree.” Higher values indicate a more pronounced self-control. In addition, the parents will also judge the self-control skills of their children as a proxy-report. Furthermore, the self-efficacy experience of the children will be assessed using an adaptation of the weight-specific self-efficacy questionnaire for children and adolescents [37]. The nine items cover the child’s confidence to deal with current temptations of sweets (for example, “How sure are you … to refrain from sweets, although they taste good?”) on a five-point scale ranging from “highly uncertain” to “very confident.” Since both methods have been adapted for the study, the psychometric properties will be assessed in a parallel sample.

In addition to these measures, we will assess weight-related quality of life and the food intake of the children. Using the weight-specific quality of life questionnaire for overweight and obese children and adolescents (GW-LQ-KJ) the weight-related quality of life will be measured. The 11 items (for example, “During the last two weeks I felt blue because of my weight”), rated on a 5-point Likert scale, reach high internal consistencies (α = 0.87) [37, 38]. Children’s food intake will be evaluated using a short nine-item food frequency list including “healthy” (vegetables and fruits) and “problematic” food items (for example, sugared drinks, salty snacks, and sweets). Children are asked to rate their usual intake on a five-point scale (“never” to “several times a day”). In a previous study (Warschburger P et al.: Empowering parents of obese children (EPOC): A randomized-controlled trial on additional long-term weight effects of a parent training, submitted) , reliability was acceptable for the “healthy food intake” scale (α = .78) but low for the “problematic scale” (α = .53). In order to increase the reliability of the latter scale, it was expanded with additional items (for example, eating fast food and sweet dairy products) for the present study.

### Predictors

Binge eating will be assessed via a child’s self-report on experiencing attacks of binge eating and psychosocial strain with the parent version of the Strengths and Difficulties Questionnaire (SDQ), a reliable and valid screening questionnaire [39].

### Randomization procedure and treatment allocation

Randomization and allocation in a ratio of 1:1 for each clinic to treatment aim will occur before the interventions begin. The randomization is computer based [40] and centrally performed. A post-hoc evaluation by descriptive statistical comparisons between the intervention and the control group in relevant sociodemographic data (age, gender, and sociodemographic data) will be conducted to assess the effectiveness of the randomized assignment. The study assistant will receive the fax with the randomization that only discloses the allocation of the patients to either group A or B (allocation concealment).

### Points of measurement

The data are collected by questionnaires and computer assessment for children at four points of measurement. Assessment of the pre-intervention (T1) and post-intervention (T2) data will be conducted at the clinic during the inpatient stay, the follow-up measurements for the 6- and 12-month follow-up at home. At the beginning (T1) and the end (T2) of the inpatient stay, the weight status (the primary outcome) is assessed by the physician in charge. Children and their parents fill in the self- and proxy-report questionnaires, respectively. The computer-assisted assessment (AAT; IAT; dot probe and Stroop) will be conducted directly before and after the last training session. Two follow-up assessments will take place, the first, 6 (T3) and the second 12 (T4) months after the [41] discharge from the clinic and at the end of the intervention. Questionnaires will be sent via mail in order to collect the self-report and proxy-report data. In addition, objective weight and height measurements will be conducted by the physician of the participant using calibrated measurement devices. In order to receive the weight-related data of the follow-up measurements, the patients are asked to grant the study center permission to contact their physician in order to achieve the relevant data. Additional information for the follow-up assessments will also be collected with the use of questionnaires respective computer tests. The follow-up assessment will also include questions regarding the utilization of additional weight-loss interventions, as this might influence the primary endpoint.

### Blinding

The following guidelines will be used to ensure that the data is blinded during intervention and assessment. The preassessment will precede the randomization and will be conducted by the study assistants on site; these study assistants will not be informed about the study goals in general and the computer training in specific. The assessment of anthropometric data will be conducted by medical staff not involved in the study and therefore blind to hypotheses and study design. The allocation of the participants will be done by fax using a mixed letter coding system. Therefore the study assistants and the participants will be blind to the treatment allocation. Data collection and data analysis will be independently conducted. A scanner is used to for the data entry. Whenever feasible, online questionnaires will be used for the collection of the follow-up data. In any case, only the code of the child will be given, which allows no conclusions about the treatment allocation. The child’s physician will provide the study center with the anthropometric data at the follow-up. The physician will also be blind to the goals of the study. Reimbursement will be used to prevent high as well as selective attrition rates. Regular unblinding will take place after the completion of the data analysis. No intention exists for early unblinding.

### Participant discontinuation and safety

Participating children and adolescents, as well as their parents, can withdraw their study participation at any time. In addition, they can discontinue treatment without giving any reasons. Since no adverse effects of such a training program were reported in the literature (for example, [42]), we do not expect any severe side effect of our treatment approach. We will document any reported negative effect from the participants of the computer training. In addition, the occurrence of disturbed eating patterns will be monitored at the follow-up assessments.

### Intervention evaluation by the participants

At the end of the AAT-training sessions, children and adolescents will fill in a self-constructed acceptance questionnaire including open questions to give the participants the opportunity to comment on their experiences. In addition, the assistants will note signs of discomfort.

### Trainer meetings and monitoring

Trainer meetings are not necessary because we are using a standardized computer training. The study coworkers will be trained on how to approach eligible participants and how to apply the computer training and the assessment sessions in a standardized way. Furthermore, they will be provided with a study manual. Therefore the standardization of recruitment, data collection and intervention assures data quality.

### Ethical approval

This trial received written approval by the ethical committee of the University of Potsdam (proposal 29/2012; vote on 05 November 2012). The study will be conducted according to the principles outlined by the Declaration of Helsinki (version 2013). The staff commits themselves to all pertinent national laws and the ICH guidelines for GCP issued in June 1996 and CPMP/ICH/ 135/95 from July 2002. All relevant parties will be informed in case of important protocol modifications.

### Data

All staff members that come into contact with the data are subject to professional secrecy and data protection conditions. Personalized data will be assessed via questionnaire and will be stored in a locked cabinet separate from the other data and not electronically filed. With respect to the reaction time, data they will be saved on the respective computer on each study site. The recordings will be copied onto a password protected CD by the study nurse and sent by mail to the study office. The access to the computer is restricted; the computer is not connected to the internet. The training sessions start after entry of the code number by the study assistant. All additional data (medical sheets and questionnaires) are marked with code numbers and pseudonymized. Following the final assessment, all personal data will be destroyed to ensure immediate complete anonymization. Before consent is given, all potential study participants receive adequate written information about the aims and implications of the study. Informed consent will be obtained from children and their parents by the study assistants.

All primary outcome data (weight data) will be verified supplementary to the electronic data entry via scanner. Additionally, 20 % of the questionnaire data will be double checked by staff. A Data Monitoring Committee will not be appointed, as risks from the intervention are neither reported nor expected.

### Data analyses

Missing data of the computer-assisted measurements will not be replaced since there are very unlikely and might be a sign of recording problems. With respect to the questionnaire data and the primary outcome, missing values will be replaced by unbiased substitution (EM-algorithm). As a first step, an analysis of whether the children in the intervention group show a learning effect compared to the control will be conducted. This learning effect would be characterized by faster reaction times at the end of the training. Primary and secondary outcomes will be tested performing an ANCOVA from post-intervention to the 12-month follow-up between the two random groups, adjusted for the baseline measurements. They will be analyzed by performing an ITT as well as a per-protocol analysis (PPA) that excludes all drop-outs. The association between self-regulation and sustained weight loss at 12-month follow-up and potential moderators (binge eating; psychosocial strain) will be tested using hierarchical multiple regression analyses. As a second step, the BMI-SDS change will be dichotomized (successful versus not successful) in order to perform logistic regression analyses. Relevant trial results will be published in the corresponding platforms.

## Discussion

In order to reduce one’s overweight in the long term and maintain a healthy weight, a comprehensive lifestyle change is necessary. These programs target mainly the change in diet and exercise behavior and require high self-regulation skills from the participants. When living in an obesogenic environment of everyday temptations of high-calorie, palatable food, one’s ability to self-regulate becomes of special importance in weight management. Recent studies provide increasing evidence that self-regulation skills are impaired among overweight and obese children, and those with lower self-regulation skills are more prone to experience future weight gain.

However, self-regulation skills are trainable, and improvement of these skills appears to be a promising approach to achieve long-term weight loss. Regular practice on the PC, with repeated confrontation with a special task seem particularly suited to build new, stable automatic patterns of behavior that oppose the “obesogenic” behaviors. Growing evidence suggests that eating behavior cannot only be considered as a deliberate decision but also as an automatic process that should be taken into account [43]. In particular, implicit training programs that merely focus on improving one’s own reaction times to particular stimuli strengthen the automatic pathway. Therefore, the children are not constantly confronted with the requirement to consciously change their behaviour – a task that will overwhelm and ultimately most often discourage them.

As we above mentioned, an approach-avoidance training seems to be a promising approach to strengthen automatic avoidance patterns. Although, no studies are available on that topic in children, some first studies, with predominantly adult participants, have been published. These studies support our hypotheses with respect to the positive effects by means of computer-based self-regulation trainings. Boutelle, Kuckertz, Carlson, and Amir [44] trained obese children in only a single session to either divert their attention from food-related words whenever presented on the screen (intervention) or only in half of the times in the control condition. The authors reported a higher caloric intake during an unrestricted food task for the children in the control group over the course of the experiment, that is, the control group increased their intake from pre- to post-intervention. However, this time effect was not observed in the intervention group. While it is relatively well documented in the literature that obese individuals have an attention bias toward food [45], now evidence also suggests an approach bias toward food [46]. These findings support that a training program based on the approach-avoidance paradigm may represent a potential target for interventions. Corresponding trials for children and adolescents are not yet available. However, in various studies with adults, the consumption of snacks has been reduced by strengthening the inhibitory control by means of a go/no-go training [42, 47–49]. Verbeken, Braet, Goossens, and Oord [50] trained the general executive functions and working memory of obese children within an inpatient treatment over 25 sessions. Compared to the untrained control group, children of the intervention group were better able to maintain their achieved weight loss at the end of the inpatient treatment. This effect was observed only 8 weeks post-intervention but was no longer significant at the 3-month follow-up. Overall, these studies could underscore that the improvement of self-regulatory skills can have positive effects on the eating habits, as well as on weight development. However, so far, little to nothing is known about the long-term effects and putative mediating mechanisms.

In addition, computer-based training programs in general are often considered as an attractive approach and may be accompanied with an increased adherence compared to “traditional approaches,” especially in children and adolescents. Such a program can easily be used as an additional ingredient in multimodal lifestyle programs. Furthermore, computer-based training programs are suitable as a web-based follow-up training because the children can independently complete the training sessions similar to a computer game. Especially, the use of this program as a follow-up training seems attractive, as long-term maintenance of treatment effects is the biggest challenge in weight-loss interventions.

## Trial status

Recruitment started in March 2015 and is still ongoing.

## Abbreviations

AAT: Approach-Avoidance Training
ANCOVA: analysis of covariance
BMI: body mass index
BMI-SDS: standardized BMI-score
BSCS: Brief Self-Control Scale
GW-LQ-KJ: weight-specific quality of life questionnaire for overweight and obese children and adolescents [Fragebogens zur gewichtsbezogenen Lebensqualität für übergewichtige und adipöse Kinder und Jugendliche]
IAT: Implicit Association Test
ITT: intention-to-treat approach
PPA: per-protocol analysis
SDQ: Strengths and Difficulties Questionnaire
SRT-Joy: Self-Regulation Training Joystick
